# Bringing Excitement to Empirical Business Ethics Research: Thoughts on the Future of Business Ethics

**DOI:** 10.1007/s10551-022-05242-7

**Published:** 2022-09-15

**Authors:** Mayowa T. Babalola, Matthijs Bal, Charles H. Cho, Lucia Garcia-Lorenzo, Omrane Guedhami, Hao Liang, Greg Shailer, Suzanne van Gils

**Affiliations:** 1 Nazarbayev University Graduate School of Business, Nur-Sultan, Kazakhstan; 2 RMIT University, Melbourne, Australia; 3 Lincoln International Business School, University of Lincoln, Lincoln, UK; 4 Schulich School of Business, York University, Toronto, Canada; 5 The London School of Economics, University of London, London, UK; 6 Darla Moore School of Business, University of South Carolina, Columbia, USA; 7 Lee Kong Chian School of Business, Singapore Management University, Singapore, Singapore; 8 College of Business and Economics, Australian National University, Acton, Australia; 9 Department of Communication and Culture, BI Norwegian Business School, Oslo, Norway

**Keywords:** Psychology, Leadership, Methodological innovation, Finance, Accounting, Paradigms, Social relevance, Future of business ethics

## Abstract

To commemorate 40 years since the founding of the Journal of Business Ethics, the editors-in-chief of the journal have invited the editors to provide commentaries on the future of business ethics. This essay comprises a selection of commentaries aimed at creating dialog around the theme *Bringing Excitement to Empirical Business Ethics Research* (inspired by the title of the commentary by Babalola and van Gils). These editors, considering the diversity of empirical approaches in business ethics, envisage a future in which quantitative business ethics research is more bold and innovative, as well as reflexive about its techniques, and dialog between quantitative and qualitative research nourishes the enrichment of both. In their commentary, Babalola and van Gils argue that leadership research has stagnated with the use of too narrow a range of perspectives and methods and too many overlapping concepts. They propose that novel insights could be achieved by investigating the lived experience of leadership (through interviews, document analysis, archival data); by focusing on topics of concern to society; by employing different personal, philosophical, or cultural perspectives; and by turning the lens on the heroic leader (through “dark-side” and follower studies). Taking a provocative stance, Bal and Garcia-Lorenzo argue that we need radical voices in current times to enable a better understanding of the psychology underlying ethical transformations. Psychology can support business ethics by not shying away from grander ideas, going beyond the margins of “unethical behaviors harming the organization” and expanding the range of lenses used to studying behavior in context. In the arena of finance and business ethics, Guedhami, Liang, and Shailer emphasize novel data sets and innovative methods. Significantly, they stress that an understanding the intersection of finance and ethics is central to business ethics; financial equality and inclusion are persistent socio-economic and political concerns that are not always framed as ethics issues, yet relevant business policies and practices manifest ethical values. Finally, Charles Cho offers his opinion on the blurry line between the “ethical” versus “social” or “critical” aspects of accounting papers. The Journal of Business Ethics provides fertile ground for innovative, even radical, approaches to quantitative methods (see Zyphur and Pierides in J Bus Ethics 143(1):1–16, 10.1007/s10551-017-3549-8, 2017), as part of a broad goal of ethically reflecting on empirical research.

## Bringing the Excitement Back into Research on (Un)Ethical Leadership

**Mayowa T. Babalola and Suzanne van Gils**

**Introduction**

We, the co-editors of the Leadership and Ethics: Quantitative Analysis section, would like to use this occasion to congratulate the Journal of Business Ethics on its 40th anniversary. Compared to the long history of topics in business ethics, systematic research on ethical leadership only began to gain traction in the early 2000s—with some exceptions (e.g., Treviño, 1992). Since then, this area of research has grown substantially, with most research building on Brown et al.’s (2005) definition of ethical leadership as “the demonstration of normatively appropriate conduct through personal actions and interpersonal relationships, and the promotion of such conduct to followers through two-way communication, reinforcement, and decision-making” (p. 120). Related leadership concepts such as authentic leadership, servant leadership, and respectful leadership have also gone through similar development trajectories, establishing relationships to outcomes such as organizational citizenship behavior, performance, and counterproductive work behavior, mediating mechanisms such as social learning and exchange, and personality or situational-based antecedents (Hoch et al., 2018).

While extant research in this domain to date has yielded valuable insights, we argue that research on leadership ethics has reached a point of stagnation when it comes to *novel* insights and that it may be time to bring the excitement back into the research on ethical leadership. In this commentary, we discuss our ideas on how this could be established and highlight recently published articles in the Journal of Business Ethics that could serve as examples.

In this commentary, we highlight some critical issues stifling the advancement of research on (un)ethical leadership. First, we discuss the need for critical disentangling or integrating of ethical leadership styles. Second, we suggest adopting innovative methodological approaches to assess constructs in the (un)ethical leadership domain. Third, we discuss ideas for extending the current understanding of (un)ethical leadership by exploring outcomes relevant to business and society and new trends in digital leadership and ethics. Finally, we emphasize the need to acknowledge different perspectives in the study of leadership ethics—on the one hand, by integrating the role of culture, follower influence, and the potential dark sides of ethical leadership, and, on the other, by reversing the lens and focusing more on follower influence. To conclude, we offer some tips and recommendations to authors aiming to advance the field of ethical leadership. In sum, this commentary offers guidance to authors on the kind of papers that will bring back the excitement into (un)ethical leadership research and provide significant learning opportunities for practitioners.

### Critical Disentangling or Integrating of Ethical Leadership Styles

The field of ethical leadership is currently facing heavy critique for conceptual conflation and high intercorrelations between the different leadership styles (see Banks et al., 2021). Meta-analyses and reviews, on the one hand, advocate for a better definition and disentangling of the ethical leadership styles (Banks et al., 2021), and, on the other, show little incremental value of one style over the other (Hoch et al., 2018). Meta-analyses that provide critical suggestions on how the different leadership styles are similar or distinct (Banks et al., 2021; Hoch et al., 2018) are crucial for the advancement of the field. In this regard, we also repeat the call by Palanski et al. (2021) for studies that go beyond the usual conceptual suspects and perhaps even combine different leadership behaviors. Research on abusive supervision, defined as leaders’ hostile verbal and nonverbal behaviors, excluding physical contact (Tepper, 2007), could be an example. While abusive supervision has at times being treated as the opposite to ethical leadership, the literature has mostly discussed it as an independent construct with unique antecedents, outcomes, and related processes. Recent studies in this domain have addressed an increasing variety of antecedents and psychological effects (e.g., Almeida et al., 2021). While such approaches have been suggested for the domain of ethical leadership (Den Hartog, 2015), only a few studies have investigated these topics.

### Bringing the Excitement Back Through Innovation of Research Methods

The field has also been criticized for its predominant reliance on survey research methods (Banks et al., 2021). This is, in part, because survey research limits the understanding of actual organizational dynamics. In this respect, novelty and excitement in the domain of concept clarity may come from studies that focus on ‘what leaders *actually* do’—investigating leader behaviors by using interview techniques (Haar et al., 2019), archival or big data, and experimental intervention studies (Palanski et al., 2021).

### Bringing the Excitement Back Through Societally Relevant Research Questions

Another possibility for developing new and exciting research on leadership ethics is to focus on outcomes relevant to issues central to business and society today. Large numbers of studies have focused on a limited set of outcomes that put organizational performance center stage (e.g., employee ethical behavior, organizational citizenship behavior, and counterproductive work behavior; Banks et al., 2021; Den Hartog, 2015). In contrast, many fewer studies have focused on the effects of leadership ethics on outcomes that benefit individuals or society as a whole, such as employee well-being or stress-related outcomes (Vullinghs et al., 2020). While there is a wide range of potentially interesting outcomes that leadership ethics may influence, we argue that research on leadership and ethics is more suitable to address current societal challenges and questions. For example, what is the role of ethical leadership in addressing climate change? Or what is the role of ethical leaders in communities facing war, terrorism, and other life-threatening events? More research is also needed to understand how ethical leadership can be applied in digital and remote settings, as experienced during the Covid-19 crisis (Carsten et al., 2021). In addition, as work is increasingly digitalized and involves interaction with human as well as non-human actors, ethical leadership in the current age may involve a range of new dilemmas spanning from management of digital teams to monitoring of artificial intelligence and social media interactions. By addressing these questions, researchers will further advance our theoretical knowledge of ethical leadership and offer more valuable recommendations that meaningfully impact business and society and inform public policy.

### Bringing the Excitement Back Through Acknowledgment of Different Perspectives

In addition to methodological innovations and societally relevant outcomes, new and exciting insights into ethical and moral leadership may also be derived from acknowledging different personal, philosophical, or cultural perspectives. For instance, a much-discussed but less addressed perspective lies in the domain of cultural and philosophical diversity of ethical perspectives. Various reviews have pointed to the domination of Kantian and WEIRD (Western Educated Individualist Rich Democratic) perspectives in the field of moral leadership (e.g., Eisenbeiss, 2012). Although many studies are submitted to the Journal of Business Ethics that rely on non-Western populations (e.g., Wang et al., 2021), those studies rarely propose extensions of ethical (leadership) theory through the integration of local cultural values or philosophies. This is unfortunate, because in particular values related to moderation (vis-à-vis indulgence), respect for tradition or ascription-based status that are central to the Buddhist or Islamic religion, or virtue ethics in general, are currently underrepresented in the Western-oriented definitions of ethics. One example of extension of research on business ethics through integration of cultural values can be found in recent research by Haar et al. (2019). They utilized the context of the Māori culture to show the importance of values such as humility, altruism, and cultural authenticity for ethical leadership perceptions. These concepts are central to the philosophy of ethics, but have not necessarily been discussed in depth in the context of (un)ethical leadership before. Thus, we suggest that there may be valuable insights gained from extending the current operationalizations of ethical leadership constructs and rethinking existing research models through the integration of values and philosophies that may be more central populations that are marginalized (Alm & Guttormsen, 2021) and in settings that include different cultural perspectives (Palanski et al., 2021). Future studies that integrate local cultural values into their theoretical model may be particularly insightful in guarding the field’s epistemic diversity and in helping to challenge some of our implicit assumptions on (un)ethical leadership.

### Bringing the Excitement Back by Reversing the Lens

We also echo previous calls to look at the other side of the coin of ethical leadership by either taking a follower perspective or acknowledging that there may be negative effects of positive leadership (Palanski et al., 2021). Research that focuses on the influence of followers on ethical leadership can contribute to this theorizing, for example, by discussing when followers deviate from a course of action outlined by ethical leaders (Uhl-Bien & Carsten, 2007), or by discussing exceptions to positive effects of ethical leadership for some followers (Wang et al., 2021). Regarding the negative effects of ethical leadership, Stouten et al. (2013) showed that *too much* ethical leadership demotivates follower citizenship behavior. More research is needed to enhance further our understanding of the unintended negative effects of ethical leadership, and this may also help to rethink the necessary conditions for leadership to qualify as “ethical.”

Finally, numerous research studies have studied unethical forms of leadership from an actor-centric perspective or with a focus on temporal or daily dynamics (Liao et al., 2021). Such research is limited in the domain of ethical leadership (see Lin et al., 2016, for an exception). In their study, Lin et al. (2016) found that leaders’ displays of ethical behaviors were positively associated with abusive behaviors the following day via ego depletion and moral credits from their earlier ethical behavior. This finding points to the need for more research on the potential costs and benefits of ethical leader behavior for leaders themselves. Research adopting an actor-centric perspective will help further clarify whether and under what conditions ethical leader behavior is beneficial versus detrimental to leaders’ own well-being. We would also like to encourage researchers to devote more attention to uncovering the dynamics and antecedents of ethical leadership in organizations. If ethical leadership promotes beneficial outcomes (Brown et al., 2005), why is this leadership behavior not embraced as expected?

**Conclusion: Five Tips**

Concluding this commentary, we suggest that authors may promote new excitement in the domain of leadership ethics through the following five tips:

1. Aim to advance the field through critical disentangling or integrating of ethical leadership styles;
2. Use new methods (experiments, big data) to investigate what leaders actually do;
3. Focus on societally relevant questions and outcomes, addressing the ethical challenges that business leaders face today;
4. Extend theorizing on ethical leadership by integrating marginalized perspectives and promoting cultural and philosophical diversity; and
5. Reverse the lens by investigating whether and how followers’ actions can facilitate or enhance ethical leadership practices in organizations.

## Future of Psychology and Business Ethics

**Matthijs Bal and Lucia Garcia-Lorenzo**

**Introduction**

We desperately need radical voices in these current times to enable a better understanding of the psychology underlying ethical transformations. Previous calls in the Journal (Islam, 2020) have already outlined the multilevel approach required to develop an engaged and current approach to a Psychology of Business Ethics that considers individual psychological behavior within the social, historical, and organizational contexts where it occurs. However, most work on the psychology of business ethics still shares a primarily quantitative micro focus on a single ethical variable (as IV or DV), presents mediation/moderation models trying to predict unethical behaviors in organizations and assumes that unethical behaviors are only those behaviors harmful to organizational performance. While some of these are relevant and interesting, too often papers do not really engage with the deeper issues underpinning the psychology of business ethics. We reiterate calls for building the future of this section, and, more broadly, the field of psychology and business ethics, with papers that have a greater emphasis on understanding the psychology of individual and group behavior, attitude, feeling, cognition, and decision making within the context where it is performed (cf. Islam, 2020). Understanding the psychology of ethical behavior in societal contexts will also allow the field of psychology to disengage from its tainted past (e.g., racism: APA, 2021) to radically redefine itself going beyond serving corporate or hegemonic interests (e.g., Bal & Dóci, 2018). Psychology can support business ethics by not shying away from grander ideas, going beyond the margins of “unethical behaviors harming the organization” and by expanding the range of lenses used to studying behavior in context, including more constructivist, performative, and processual approaches. Methodologically this would require an expansion of ways of looking beyond simple quantitative models used for prediction of specific behaviors with specific predictors. Ethical behavior cannot be merely explained based on a micro psychology focused on reproducing the status quo.

Any meaningful psychological research needs to understand human beings as embedded within the broader context of organizations, society, and ideological manifestations. Despite recent calls in the Journal to expand and deepen our understanding of the link between business ethics and psychological processes; creativity, critical thought, or imagination are often rather absent in the field. As a result, the standard Journal articles focusing on unethical behavior hardly contribute anything significant to the understanding of the psychology of business ethics. Only a marginal part of the total set of submissions dare to focus on interesting topics (e.g., prejudice, harassment, or moral reasoning), or apply interesting methodological approaches that shed new lights and perspectives on trite topics in the field of business ethics and psychology (e.g., through using qualitative methods, discourse analysis, or conceptual methods). The scarcity of these submissions, however, raises significant questions about the state of the art concerning psychology and business ethics.

Our approach follows and complements Islam’s (2020) call for a multilevel approach to structure research on the psychology of business ethics. We build on his framework to focus on explaining how such a framework may be translated into submissions to the section on Psychology and Business Ethics. Building on the multilevel model moving from societal contexts to intrapsychic contexts to explain the psychology of business ethics, scholars in our field can make meaningful contributions to the literature. First and foremost, we want to emphasize that publishing in academic journals should not be the sole focus of an academic career, despite the pressures scholars face as universities demand their scientists publish in (top tier) scientific journals to be able to secure a career in academia. While we are aware of the pressures, we would like to support the structural change required to turn the tide on the increasingly uninteresting, incremental, risk-avoiding research that has come to fill contemporary work psychology, management, and business ethics journals. Publishing on the topic of Psychology and Business Ethics should be rediscovered again as an endeavor to provide a meaningful contribution to our understanding of the broad topic of psychology and business ethics. We discuss in the following piece the ways through which we, as a community of psychologists interested in business ethics, can write papers that are more meaningful, relevant, and interesting to read for other scholars.

### The Future of Psychology and Business Ethics

Too much research within the psychology of business ethics has implicitly adhered to the instrumental logic underpinning human behavior in the workplace (Bal & Dóci, 2018). In this logic, humans are instrumental to organizational performance and profit, and behavior not aligned with organizational goals is perceived as *unethical*. This is a somewhat surprising underpinning of work on the psychology of business ethics, as the American Psychological Association (APA) ethical code (a psychologist’s ethical code gold standard) clearly defines the duty of psychologists to respect and protect the dignity of human beings (APA Code, 2017, Principle E). It is far from evident that organizational interests are aligned with the principle of human dignity, and a more critical engagement with such issues is necessary in the current era of neoliberal capitalism (Shymko & Frémeaux, 2021). Accordingly, business ethics has been hijacked too often as a tool for the improvement of organizational performance, based on the (mis)understanding that “more” ethics means better performance (e.g., Goebel & Weißenberger, 2017). For obvious reasons, this is a rather contested notion, as ample evidence shows that organizational *un*ethical behavior may constitute the main basis for profit. For instance, the destruction of the planet by energy and fossil fuel companies creates a basis for enormous profit, and there are serious questions to be asked about the ethics of individual and collective behavior in such organizations. Merely assessing whether these behaviors are counterproductive to organizational goals constitutes an insufficient perspective on the psychology of business ethics because it ignores the context in which such behaviors emerge (see Islam, 2020). What could or should research do to make meaningful contribution to the psychology of business ethics? In what follows, we discuss issues relevant to the development of greater understanding of this field.

### Psychology Inherently Integrated in Context

Research that truly aims to make a contribution to understanding of the psychology of business ethics needs to consider the individual with or in the context, and thus, ethical behaviors, attitudes, feelings, cognition, and decision making (Islam, 2020) can only be properly understood when the context in which they emerge, are maintained or rejected is also explored. Thus, research on the psychology of business ethics needs to move beyond its current focus on individual rationality and revisit the understanding that ethical behaviors and morality are based on the interdependence between the self and other(s) as an ontological (existential) point of departure. Individual and relational rationality are two different forms of thought which determine the kinds of questions we pose about humans and their capacities and have fundamental implications for questions about the nature of thinking and knowing, about individual and social action, as well as about ethics and morality. A relational and contextual approach to ethical behaviors affirms that humans act in order to promote what they consider as good, just and worthwhile, even if what some consider as good, just and worthwhile, others judge as misery, injustice, and worthlessness. Whatever the meaning of good, just and worthwhile, contextualized ethical behaviors based on the interaction between self and other(s) are about the fulfillment of “living” (Taylor, 2011). It was Ricoeur who emphasized the idea of ethics as “good life.” He argued for the priority of ethics, that is, of the self’s search for the “good life” with others and with or in institutions based on justice, over what is usually called normative morality. Normative morality, while indispensable in social life, must be subsumed under ethics (Ricoeur, 1990/1992). Contextualized ethical behaviors based on a self–other(s) interdependence permeate all daily thinking, communicating, and acting in organizations, and they are, therefore, of major interest to a renewed psychology of business ethics.

From this perspective, unethical behavior cannot be simply equated with behavior harming organizational interests, while the ethicality of the organization itself and its practices towards, e.g., the dignity of people and the planet (Bal, 2017) need to be also considered. To do so, it is also needed to challenge and move beyond the comfortable practices of quantitative research and the moderated-mediation model. Psychological research and practice is often projected to be value free, neutral, and objective, but there is increasing awareness of the fallacy of such assumptions, as, for instance, indicated by the acknowledgment and apology by the APA of its contribution to the perpetuation of systemic racism (APA, 2021). Hence, under the banner of “objective research” using quantitative research methods, psychology has too often fulfilled an ideological role, thereby favoring hegemonic interests in institutions, organizations and society at the expense of vulnerable groups. In light of such *unethical* past, psychology needs to engage with contemporary workplace issues in a more radical way to remedy its unethical historical legacy. Hence, fundamental questions need to be asked about the ethics of psychology in business, and what psychology has to offer society beyond the perpetuation of the status quo, and cozying up to hegemonic ideologies (e.g., Bal & Doci, 2018).

The world is currently facing a number of grand challenges, including the impact of climate change, increasing inequalities, populism, and racism. These challenges also impact on work experiences, and the role of psychology in addressing these issues is still under-acknowledged. While ethics is about the “right thing to do,” this has various implications for the psychology of business ethics: researchers in this field may focus more on the constraints and facilitating factors that contribute to individual attitudes, decision making and behavior in the context of these grand challenges of society. It is well established that more individuals in workplaces want to contribute to a more sustainable world and more sustainable workplaces, and the psychology of how such individual motives can be translated into meaningful action towards more sustainable organizations and societies is currently under-researched. Research in the field of the psychology of business ethics may play a meaningful role in assessing how individual behavior may contribute to addressing the grand challenges of global society, as well as assessing how individuals make sense of these grand challenges, and how this impacts upon their lives. As business ethics is about what constitutes the right thing to do in the context of work, individual behavior is ultimately key to understanding this process. Hence, we would like to call for a revaluing of psychology as the understanding of how individuals behave in the context of business ethics and grand societal challenges, such as the Sustainable Development Goals.

Psychology is currently in a state of crisis, whereby its legitimacy and meaning are questioned by both scholars and practitioners (Highhouse et al., 2020). In response to the legitimacy crisis of psychology, and the scarcity of responses generated by psychologists to great challenges of today, including increasing inequalities, climate change and polarization in society, it may be not so much a question of psychology becoming *too* radical, but of psychology *not being radical enough*. We, therefore, call upon psychologists to not shy away from asking questions about the bigger context, the bigger picture, to pose more critical questions about the state of psychology and its possible contribution to the understanding of the great challenges of today’s world and workplaces, and to question the hegemonic practices that may hinder scientific progress in our field.

### Practical Recommendations

The psychology of business ethics goes beyond the study of micro-behaviors of individuals in organizations, disconnected from its broader context. We would welcome papers that attempt to scrutinize the bigger picture, and try to understand how individual behavior is shaped by societal contexts, and how individuals may contribute to the necessary transformation of business and society towards a “good life,” a more sustainable society and economy, while protecting the dignity of people and planet. Such bigger questions might be squeezed into moderated-mediation models, but they could only offer a simplified version of reality that is inherently limited to the extent that variables are captured into a model, which by definition means a selection of some variables at the expense of potentially other important factors. Instead, psychology is in desperate need of more diversity in ontological and epistemological frameworks to guide our research. Research that moves beyond the all too familiar moderated-mediation model to address bigger questions of today’s world would be most welcome for the Journal. Processual and performative approaches that study how ethical processes emerge from a psychological perspective would shed more light on contemporary issues. This way, more understanding can be generated about how unethical behaviors emerge and become consolidated and taken for granted. Research could also investigate how unethical behavior is performed in daily life in actual workplaces. Observational research would be a useful method to achieve such aims, and would force psychologists to “move out of the lab into the real world,” to assess what is actually happening in contemporary workplaces, and what the ethical dimensions of contemporary workplaces are.

**Conclusion**

Thus, we call for a psychology of business ethics that supports and promotes engagement with real world problems and aims to have an impact on the type of change necessary for sustainable development and well-being.

## Business Ethics Issues in Finance: Challenges and Recommendations

**Omrane Guedhami, Hao Liang and Greg Shailer**

**Introduction**

Public interest in the ethical aspects of finance and related behaviors and activity is reflected in the growing number of submissions to the Finance and Business Ethics section at the Journal of Business and Ethics (JBE). Our objective in the following discussion is to provide a better understanding of the domain of Finance and Business Ethics by first describing some recurring issues with submissions to the Finance and Business Ethics section. We then provide some guidance as to where we see opportunities for meaningful contributions to the finance-ethics literature. This includes advocating for more diversity in empirical methods and the use of novel data, and identifying some areas we think are important but relatively under-researched.

In this commentary, we highlight the importance of combining innovative and rigorous analysis with fundamental economic questions when studying the intersection between business ethics and finance. We first outline some major issues associated with conducting research on ethical issues in finance, and then discuss possible directions in which finance and business ethics research might be further developed and integrated; this includes encouraging methodological diversity and identifying research areas we believe are important but under-researched.

### Challenges in Conducting Research on Finance and Business Ethics

#### Relevance to Scope

To provide a substantial contribution to knowledge at the intersection of business ethics and finance, a study might offer new insights that advance our understanding of ethical issues in finance, or use knowledge or expertise from the finance field to contribute to a larger conversation about ethics. A major issue we encounter when reviewing manuscripts submitted to our section is that many do not offer sufficient contribution to our knowledge of business ethics. Many such studies lack a genuine ethics dimension—with any reference to ethics being largely gratuitous—and a sound theoretical framework and hypothesis development that is grounded in the business ethics literature.

As section editors, we observe two noteworthy types of submissions to the Finance and Business Ethics section that exhibit one or more of the above deficiencies. The first type typically relies on the finance literature and theories, while omitting a persuasive reference to the business ethics literature. For example, a substantial number of rejected submissions have adopted an agency perspective to examine the relations between ownership structure (e.g., family, government, employee, institutional) and corporate financial performance (e.g., profitability, valuation, cost of capital) or outcomes (e.g., payout policy, investment, risk, capital structure, CEO compensation, CEO turnover). Other examples include examining aspects of risk taking by financial and non-financial corporations, assuming that it is detrimental to stakeholders, and examining the determinants or implications of gender diversity for corporate outcomes. It is accepted that agency and ownership issues, risk-taking behaviors, and diversity issues *can* be meaningfully examined from an ethics perspective, but this does not mean such topics are inherently concerned with or contribute to knowledge in ethics, and it is incumbent on authors to persuasively establish the ethical dimensions or relevance of the study. Even when topics studied have some obvious broad connections with business ethics (e.g., earnings manipulation, fraud, tax avoidance, treatment of employees, greenwashing, pollution), the ethical analysis or ethics implications are usually not sufficiently developed, and the discussion of findings and their implications is largely focused on performance or risk consequences. In possibly gratuitous attempts at satisfying JBE’s scope requirement of business ethics relevance, some manuscripts sporadically include the term “ethics” (often concentrated in the introduction and conclusion), or tangentially refer to studies on related topics that were previously published in JBE.^1^ This does not bring a manuscript within the scope of JBE’s editorial objectives.

The second noteworthy scope-related deficiency is observed in submissions that largely exclude meaningful and contextualized theory reviews or development, thus, failing to present a theoretical framework and hypotheses that are grounded in the business ethics literature; usually, they instead emphasize datasets and research design. For these submissions, we observe that the contributions largely focus on the finance literature or empirical methods, with no meaningful engagement with the business ethics issues. The motivation (introduction) section of some of these submissions does not develop any theoretical arguments, and the background literature relates primarily to finance. Examples of studies exhibiting these deficiencies include some that merely compare the performance of socially responsible or Islamic stocks and funds to conventional stocks and funds, or the performance of socially responsible investing during times of crisis. While some of these papers may rely on novel data or sophisticated portfolio construction approaches, the link to business ethics is not sufficiently established and the theoretical development lacks the ethical framing or rigor appropriate to this journal.

#### Originality and Contribution

The Finance and Business Ethics section aims to publish original work that has the potential to be influential or have high impact in the domain of business ethics, while intersecting in some way with finance. In our review of submitted manuscripts, we encounter many that do not offer a sufficient contribution that meets the threshold for JBE. Some are of high quality in terms of word craft and empirical execution and satisfy the business ethics relevance requirement, but essentially replicate prior studies, using updated or expanded samples or different countries. Replications can be useful and provide additional insights into previously studied ethical issues in finance, but they do not necessarily contribute sufficient new insights if the motivation is not convincing, the theoretical development is weak, or the results are intuitively obvious or merely confirm existing evidence. To make a contribution with a different sample, one needs to carefully consider and elaborate on how such a setting offers some particular institutional features that allow the study of important business ethics questions that prior studies were not able to address.

In our view, studies exhibiting high-quality execution and writing, but insufficient originality and contribution to the domain of finance and business ethics, include those that examine financial market participants’ assessments of CSR practices, and their implications for other corporate policies, corporate performance, or risk. In addition to the challenge of establishing whether such studies exhibit sufficient relevance to business ethics, the literature addressing these issues is large and relatively mature.

#### Rigor in Empirical Execution and Analysis

In evaluating the design and execution of any analysis, we emphasize its quality and rigor. Most submissions to the Finance and Business Ethics section involve empirical analysis, with a variety of qualitative and quantitative approaches. Here, we outline some of the more common empirical weaknesses that can be avoided. We also note that studies with substantial weaknesses in their empirical design (including the extent of their ethical focus) or execution are also more likely to exhibit other substantial problems.

Our suggestions here should be read as indicative and not as a checklist. First, many submitted papers that we encounter lack sufficient transparency with respect to sample selection and characteristics. A good paper will provide a sufficiently detailed description of the sample selection process. This might include explaining the desirability of novel sampling strategies or justifying why a sample period stops earlier than appears necessary. Second, and related to the preceding point, a paper should include appropriate descriptions of sampling distributions (e.g., by industry and year) to give readers important information they can use when assessing the study design and results. Third, while innovations or improvements in empirical design are encouraged, they must be rigorously developed and justified, rather than appearing ad hoc*.* Fourth, as we discuss below, reported analyses should seriously address endogeneity issues.

Many relations within and between firms’ characteristics, behaviors, performance, and environments are inherently endogenous. This is common across many analyses in the business and applied economics domain. Here, we emphasize how important it is that future studies address these issues in a rigorous manner. It is reasonable to provide a clear discussion of the sources of endogeneity threats (e.g., omitted variables, reverse causality, measurement errors) that are specific to the reported study, use suitable methods to address the identified concerns where feasible, and report the appropriate diagnostic tests associated with each approach (e.g., tests of the validity of instruments, tests of overidentifying restrictions). Endogeneity issues are particularly critical in research that examines the direct or moderating effects of firm characteristics (e.g., CSR actions or performance, corporate governance features) that are reasonably theorized (or known) to be endogenously determined with the dependent variable or with other explanatory variables; in the latter case, this may also give rise to multicollinearity concerns. These issues threaten the validity of the modeling, not merely the interpretation and generalizability of results. Finally, we suggest that subjecting the main evidence to substantive sensitivity checks should be a common practice to reduce the likelihood that findings attributed to ethical decisions or practices do not have other credible explanations.

### Future Directions

We do not seek to present a detailed research agenda for the finance–business ethics domain but offer some thoughts regarding potentially fruitful areas that researchers might consider. We do this in two stages. First, we comment on the potential for exploiting new or novel data and innovations in analytical tools, with some general examples. We then describe some areas that we believe offer opportunities to shed new light on ethics in relation to finance.

#### Novel and Innovative Empirical Developments

Strategic attempts to develop publications based on novel datasets or innovations in analytical methods face some challenges. Novelty and innovation are not, by themselves, necessarily interesting or meaningful. The value of novelty and innovation in datasets or methods arises from the extent to which their use advances knowledge in a particular field: they might be better regarded as improved “tools,” and their use is not the contribution. If using a new dataset or an advance in econometrics merely provides confirmation of previously accepted findings, it will be difficult to establish sufficient contribution. If a novel dataset does not allow for generalizable results, the authors face the challenge of convincing reviewers and editors that the findings will be of sufficient interest to the journal’s readers. But despite these and other challenges, developments in data access and analytical methods offer significant opportunities for advancing knowledge. In identifying some of these developments below, it is not our intention to suggest specific research topics; we seek to give some general indications of means by which the literature might be advanced—but we encourage readers to consider how these might be exploited in developing some of the research areas suggested in the next part. How this might be implemented is, of course, a contextual challenge for the researcher to address.

Some innovations in empirical methods (e.g., network analysis, textual analysis) and novel datasets have emerged recently in finance and other areas of business research that are currently underexploited in advancing our knowledge of business ethics in relation to finance. Studies in business and finance are increasingly deploying datasets from other fields to identify or proxy for the effects of environmental factors, such as satellite data that can reveal urban and industrial density and development levels or pollution effects, environmental emissions data, climate data, traffic data, population health data, crime data, data from large multi-user platforms, and datasets from various forms of social media. These (often big) datasets offer potentially rich measures of factors that might motivate or influence responses to ethical issues, or might enable the identification of ethics issues from different perspectives. For example, one might infer the external acceptance of a corporation’s concern about a community’s welfare by analyzing customer feedback or the sentiment of social media messages related to the corporation.

The use of Big Data sources and methods has helped answer important questions in the finance domain that previously could not be answered using traditional archival data. For example, business and finance researchers are using machine-learning techniques to make finer or more nuanced predictions and to conduct more sophisticated textual analysis to construct more granular empirical measures or properties and discover the extent to which they can reveal otherwise difficult-to-observe behaviors, motives, or expectations and relations between them. For example, there are opportunities to follow recent studies of the language properties of earnings announcements and conference calls with analysts (such as the tone of managers’ statements or analysts’ queries, particular word usage, tenses, or other language traits) to gauge awareness of or responses to concerns about ethical issues, and perhaps then examine whether such awareness or responses are predictable or predictive of other behaviors or outcomes. Future work addressing these ethical issues in the context of finance are warranted. However, if using novel datasets and techniques involves proprietary data or sensitive information on individuals, researchers must be mindful of any limitations and potential ethical challenges this entails. Novel data sets might also introduce noise that impedes the reliable identification of effects concerning ethical decisions.

Various forms of network analysis have been used to examine peer effects on behaviors or choices in the business and corporate governance domains. These have been examined as potential consequences of diverse forms of networks, including (1) corporate networks arising from supply chain relationships, interlocking or common directorships, common shareholders, or common analyst coverage, and (2) networks as products of implied social or professional relationships between various types of decision makers. While network analysis is not new in the business domain, we think there is considerable scope to exploit this approach to investigate the extent and nature of peer effects in relation to ethics issues in the finance domain.

Some studies have used laboratory and field experiments to help with causal inference and explore the behavioral foundation of ethical decision making in finance, although historically these have faced substantial generalizability problems. A recent development here is “neural finance” studies that aim to identify the neural basis of decision making by measuring neuronal activities with the help of functional magnetic resonance imaging (fMRI) and other brain scanning techniques. This has the potential to predict outcomes from ethical decision making by observing neuronal activities that are associated with other well-documented decisions.

As a general caution at this stage, we note that research exploiting new or novel data to construct new measures must justify them conceptually and in terms of rigor. Researchers should also be aware of the potential social and ethical concerns associated with using data that involve detailed personal-level information. In the case of laboratory and field experiments, the research may even alter subjects’ pecuniary incentives and social behavior.

#### Areas for Future Research

The preceding discussion briefly considers the potential usefulness of recent developments in the availability of different forms of data and methods. In this final section, we take on the more ambitious task of identifying particular areas that we believe warrant more research attention because they may yield substantial advances in our understanding of ethics in relation to finance. Again, our commentary is brief and broad. It is only intended to encourage interest in areas we believe will help develop the finance and business ethics literature, and we do not mean to discourage work in areas we do not identify here.

The intersection of finance and ethics is a subset of business ethics, not a thing apart. The providers and users of financial products and services, and other participants in financial markets, share the contemporary responsibilities of all businesses to recognize and respond to the legitimate expectations and demands of stakeholders. Such expectations and demands include the nature and use of products and services, their engagement with and treatment of investors, employees, suppliers and customers, and environmental and social impacts. To this end, positive ethical values should be at the core of their corporate cultures, and evident in participant behavior. The ethical dimensions of behavior in this regard might overlap with legal, socio-economic, and political dimensions, but they are not the same constructs. It is important to keep this in mind when reading our comments to avoid mistakenly interpreting issues associated with social responsibilities or impacts, regulatory obligations and compliance, or political connections and effects as inherently ethics issues. Usually, ethics issues entail conflicting interests, personal dilemmas, or under-weighting harm to other stakeholders.

Financial equality and inclusion are persistent socio-economic and political concerns in both developed and developing economies. While the social and political consequences of inequality or exclusion are not always framed as ethics issues, relevant business policies and practices can manifest ethical values. Businesses can both contribute to the emergence of inequalities and exploit such inequalities (for example, when they relocate to reduce operating or regulatory costs, or by taking advantage of less informed market participants) and can mitigate such problems by advancing policies and implementing practices that facilitate financial inclusion of members of society who might otherwise be marginalized, such as minority groups and the poor. But, at this stage, we have little robust evidence of how policies and practices in the financial sector can impact equality and inclusion, or the incentives or disincentives for pursuing such goals, and the ethical values or trade-offs that are entailed in relevant decisions.

Related to the previous area, microfinance can be socially and economically vital for people in lower economic strata, particularly in developing countries, but requires the embodiment of positive ethical values in the corporate cultures of providers, and supplier confidence in the ethics of those presenting themselves as borrowers. Recent research in some ethical aspects of microfinance has been facilitated by access to novel datasets, including crowdsourcing for microfinancing, but we remain at the early stages of developing the ethics literature in this area.

Related to both the previous paragraphs, prior work in the socially responsible investment (SRI) literature has some relevance but does not necessarily address ethical issues we think are important. For example, how might we evaluate the ethics of investing in an environmentally harmful technology to provide immediate social benefits and then later seek to mitigate the harm? Or whether expected indirect social impacts of investment, such as theorized “trickle-down” effects, can be judged as ethically comparable to direct social impacts? And what ethics criteria might facilitate the comparison? What are the ethical issues confronting investors (or other stakeholders) when they weigh and compare SRI effects on winners and losers if SRI strategies also entail divestment strategies (such as the community and employment effects of withdrawing financial resources from “sin” industries, which are then directed to more socially preferred industries)? Similar questions might be asked about the ethics issues for investors in weighing other negative and positive externalities, and the extent to which investors’ ethical preferences in this regard should influence corporate decision. Relatedly, when might socially preferred financing decisions conflict with some stakeholders’ extant ethical principles, and what are the social and ethical implications of such conflicts?

There is also considerable opportunity for research that will advance our understanding of finance-related decisions that intersect with prominent topical social questions. For example, can we better inform debate about the role of financial institutions in enabling or constraining intimate partner abuse, or expectations of decision making involving cross-border financial arrangements during periods of economic, political, or civil conflict that might range from trade conflicts to wars between countries?

On a different tack, the complexities of mathematical models and tools used in risk management generally work to reduce the transparency and accountability of the decisions they facilitate, which can affect diverse groups of stakeholders. This is particularly evident in previous financial crises. But we have little knowledge of the ethical issues involved in implementing risk management strategies. Integral to this are deficiencies in our understanding of the values associated with taking and avoiding risks, and how to observe the ethical commitment of managers.

In all decision making pertinent to the finance domain, there will be conflicts between the pecuniary and ethical preferences (and other non-pecuniary preferences) of individuals, and between the preferences of managers, investors, advisers, consumers, and other stakeholders. How to identify, model, and test such differences, and consequential actions and outcomes, presents a major—but potentially very important—research challenge.

We do not offer any resolutions to the empirical challenges in investigating finance-related decisions and practices that might entail ethical dilemmas or trade-offs, but we return to our earlier comments about the continuing emergence of new data and analytical tools that more imaginative researchers might be able to exploit for this purpose.

## The Blurry (?) Line Between Accounting Ethics and Social/Critical Accounting Research

**Charles H. Cho**

**Introduction**

Over the past several years, some intrigued scholars, in particular working in the area of social and critical accounting, have—formally and less formally—raised questions about what would constitute a submission “in scope” with accounting ethics. While the answer is not as clear-cut as expected, I provide in this commentary some insights based on my own experience as an author, reviewer, and Section co-editor of the Journal.

As Section Co-Editor of the Accounting and Business Ethics section, I regularly get asked whether a specific (accounting research) paper would be a good “fit” for possible publication—and my response always relates to which extent “ethics”—be this an ethical issue, concept, theory, or even story—is central and explicit to the paper. The papers that generally come to my attention (pre-submission, or submitted) are either “easy” to assess for suitability, or lack thereof (e.g., a clear, pure “mainstream” capital markets paper that sometimes includes a few references from JBE and/or an instrumental variable such as gender for diversity to proxy for ethics), or more on the fence (e.g., a “critical” accounting paper that challenges the status quo or the current capitalist system, or a “social” one dealing with corporate social responsibility (CSR) or sustainability issues). On the latter, the “ethicality” is not always evident, which leads me to either send it back to the authors to make it more so—or reject it before review because I see no viable path. This can be challenging at times, but I do believe that, while not always hard and clear, there is still a (possibly blurry) line between what makes an accounting paper “more ethics” versus “social” or “critical”—and these are of course not mutually exclusive either. To me, it really comes down to how accounting research is portrayed in terms of paradigms.

### Paradigms in Accounting Research

In the very first class of my PhD program (*Foundations of Accounting Research*) at the University of Central Florida, we did not “dig into” accounting research papers right away. Instead, we were exposed to the first four chapters of Burrell and Morgan’s (1979) emblematic book. While their proposed model to explain paradigms could be argued to be limited, limiting, and/or outdated, I still believe that, to date, it is one that effectively best explains what “paradigms” are and why it is important to understand the (research) world through them. In particular, Burrell and Morgan (1979) introduce a 2 × 2 matrix scheme to help classify and understand sociological theories based on four major paradigms (adapted from Fig. 3.1 “Four paradigms for the analysis of social theory,” Burrell & Morgan, 1979, p. 22).

**Table:** SOCIOLOGY OF RADICAL CHANGE

Radical Humanist (Subjective)	Radical Structuralist (Objective)
Interpretive (Subjective)	Functionalist (Objective)

SOCIOLOGY OF REGULATION

#### Functionalist Paradigm (Objective-Regulation)—Individualism

This is the dominant paradigm for organizational studies, notably in accounting (mainly due to the nature of our discipline and training). Most accounting research belongs to this paradigm with a range of beliefs. Archival research in accounting using financial data(bases) sits on this side of the spectrum. In particular, positivist theory, capital markets research and valuation models are rooted in this paradigm.

#### Radical Structuralist Paradigm (Objective-Radical)—Materialism

This paradigm has a perspective of inherent structural conflicts within society that create constant change through political and economic crises. It is concerned to develop a sociology of radical change from an objectivist standpoint. In terms of research spectrum position, archival accounting research using other types of data (non-accounting) and advocating a more critical view of the world belongs here. Yet this research still utilizes secondary data and statistical models.

#### Interpretative Paradigm (Subjective-Regulation)—Collectivism

This paradigm is concerned to subjectively understand the world as it is and the fundamental nature of the social world. Interpretivists seek to understand the very basis and source of social reality, the essence of everyday world. The very broad areas of behavioral, social, and organizational of accounting research would fit well here. Examples of methodology that illustrate this research type would be surveys, questionnaires, case studies, ethnography, phenomenological studies, and some content analysis.

#### Radical Humanist (Subjective-Radical Change)—Idealism

Proponents of this paradigm are primarily interested in releasing social constraints that connect human development. It is designed to critique the status quo and tends to view society as anti-human. Sources of this paradigm are the same as the interpretivist paradigm, notably Kant, Hegel, and early Marx. However, Marx inverted the frame of reference reflected in Hegelian idealism and forged the basis for radical humanism. In essence, this paradigm is the “perfect” opposite of the functionalist view. Often referred as an anti-organizational theory, the critical research in accounting fits remarkably well into this paradigm and is greatly hosted.

### Accounting Ethics Versus Social/Critical Accounting Research

Based on the above sociological paradigms, research perspectives, experiences, and even careers can dramatically vary across accounting scholars. This is so fundamental and foundational that it shapes a researcher’s—or even a person’s—view of the world (which in turn influences one’s view of research). We know that that “mainstream” accounting research is rooted in neo-classical economics theories and primarily, if not only, focuses on the efficiency of the capital markets. That itself emanates from the functionalist paradigm, but while it may seem difficult or impossible to tease out any ethical or ethics-related issues from that “camp,” studies looking at *unique* settings or factors associated with earnings manipulation (as a non-ethical construct) could in fact constitute a potentially good fit for JBE, in my view. But I do reiterate *uniqueness*.

Interestingly, such studies may be more likely to contribute to the accounting ethics literature than some that are couched in the interpretative or even radical humanist paradigm (and note that I am not commenting on the methods used—they are irrelevant in this discussion). There is no doubt that social accounting or critical accounting research papers belong to these left-handed, subjective paradigms, but they are not necessarily accounting ethics papers—and it is acceptable, because not every social or critical accounting paper must be an accounting ethics paper. Admittedly, there was a period where JBE did publish research (including mine!) that provided evidence of greenwashing practices (in other words, companies lying in their reports)—but the Journal’s editorial aims and scope have changed—rightfully so—to put ethics back to its core (see the last editorials). I believe that accounting ethics research is, or at least needs to be, a more subtle and “grey area” than greenwashing or CSR or emancipatory issues. There are other wonderful “homes” for this research such as Accounting, Accountability & Auditing Journal, Accounting Forum, Critical Perspectives on Accounting, Social and Environmental Accountability Journal or Sustainability Accounting, Management and Policy Journal. I also see JBE as a specialized journal, in the same way we have some journals for taxation, auditing, or information systems (or CSR). The *ethics* must be there, almost omnipresent, explicit, and central.

I will end by quoting the following superbly crafted comments from Lennard and Roberts (forthcoming, p. xx) about the challenges of undertaking CSR research (hence part of social accounting research) from an ethical frame:

First, accounting researchers need to make the explicit decision to study [CSR] issues from an ethical perspective. Making the decision to study [CSR] issues does not automatically default to being a study of accounting and business ethics. A substantial amount of accounting research dealing with [CSR] issues study their instrumental effectiveness in improving financial performance or some derivative of financial performance such as a lowering of the cost of capital, averting costly regulation, or avoiding reputational harm.

If an accounting researcher is interested in studying the ethics of [CSR], then the theory driving the study should have an ethical grounding. This leads to a second challenge—finding a […] theory [grounded in the ethics literature] that matches well with the research question under study. For example, researchers are often interested in how corporations’ [CSR] performance affects their stakeholders. Stakeholder theory is not necessarily an ethical frame from which to investigate a research question. There are several streams of stakeholder theory, including instrumental stakeholder theory, intrinsic stakeholder theory, and feminist stakeholder theory. Further, there are many ethical theories beyond just the two that have been maintained in extant accounting ethics (i.e., utilitarianism and deontology) which may be crucial in furthering ethical research on [CSR], yet researchers must acknowledge there is significant responsibility when prescribing ethical theories to scientific studies. A major concern when studying business ethics is that ethical theories may conflict, and researchers must make decisions on how to deal with these conflicts.

The third challenge for accounting researchers interested in undertaking [CSR] ethics research is specific to those wanting to do large sample empirical research. It is growing increasingly difficult to empirically test ethical theories using large sample, cross-sectional methodologies.

As highlighted and perfectly summarized by Lennard and Roberts, social accounting research (and the same applies for critical accounting) is not a by-default synonym with research in accounting ethics. Though these terms—ethics, CSR, sustainability—are often put together under one “umbrella” or in one “basket,” there are boundaries and I believe the role of the Accounting and Business Ethics section of JBE, at least part of it, is to keep a close eye on this blurry (?) line and those boundaries.

## Footnotes

1 In earlier years, JBE accepted some manuscripts that were essentially tangential or peripheral to the ethics domain, including many relating to CSR and environmental impacts, but the journal has ceased this practice in a return to its core mission of advancing knowledge in relation to business ethics, as signaled in a series of editorials that started with Greenwood and Freeman (2017, 2018). To appreciate the journal’s return to its intended domain, authors should carefully read the Aims & Scope statement on the journal’s homepage and editorials published in JBE.
